# Complete Genome Sequence of *Burkholderia cenocepacia* CR318, a Phosphate-Solubilizing Bacterium Isolated from Corn Root

**DOI:** 10.1128/genomeA.00490-17

**Published:** 2017-06-08

**Authors:** Filip Zekic, Brian Weselowski, Ze-Chun Yuan

**Affiliations:** a Department of Microbiology and Immunology, Western University, London, Ontario, Canada; b London Research and Development Centre, Agriculture and Agri-Food Canada, London, Ontario, Canada

## Abstract

Here, we report the complete genome sequence of the phosphate-solubilizing bacterium *Burkholderia cenocepacia* CR318, consisting of three circular chromosomes of 3,511,146 bp, 3,097,552 bp, and 1,056,069 bp. The data presented will facilitate further insight into the mechanisms of phosphate solubilization and its application for agricultural and ecological sustainability.

## GENOME ANNOUNCEMENT

Phosphate is critical to nearly all plant metabolic processes and functions. Organisms of the genus *Burkholderia* are known to solubilize organic and inorganic phosphate, increasing availability for root uptake (1, 2). *Burkholderia* spp. also present a multitude of biocontrol mechanisms, including siderophore production and antimicrobial activity (3, 4). Such organisms, termed plant growth-promoting rhizobacteria (PGPR), can positively contribute to the development of host plants and are attractive candidates for use as biofertilizers and biopesticides (5). However, strains of *Burkholderia* have been found to colonize human sputum and cause infections within the lungs of cystic fibrosis patients (6, 7). Due to a lack of insight about the molecular and genomic distinctions between PGPR and human-pathogenic strains, heavy regulations are in place on the commercialization of *Burkholderia* spp. in agriculture (8).

*Burkholderia cenocepacia* CR318 was isolated from corn root in an agricultural soil sample in London, Ontario, Canada, according to Weselowski et al. (9). A single colony was grown in nutrient broth for 24 h at 30°C, and its DNA was isolated using a GenElute bacterial genomic DNA kit (product no. NA2120), as specified by the manufacturer’s protocol. A genomic library with average insert size of 500 bp was prepared for sequencing using a Nextera XT DNA library preparation kit (Illumina, Inc., San Diego, CA, USA) and used as the template for limited-cycle PCR, as specified by the manufacturer’s protocol.

Sequencing was performed at 100× coverage using the MiSeq platform (ACGT, Inc., Wheeling, IL, USA), generating 7,990,839 raw read pairs. Reads with overlapping sequences were assembled *de novo* into contigs using SPAdes version 3.50 (10), resulting in 98 contigs ranging from 132 bp to 2.8 Mb. The final contig assembly was aligned to the reference genome *B. cenocepacia* HI2424 (accession no. GCA_000203955.1) using Mauve (11) to produce a physical genome map. PCR and Sanger sequencing were performed to close all gaps between contigs.

The complete genome of CR318 comprises three circular chromosomes of 3,511,146 bp (G+C content of 66.71%), 3,097,552 bp (G+C content of 66.82%), and 1,056,069 bp (G+C content of 67.32%). Annotation was completed using the NCBI Prokaryotic Genome Annotation Pipeline (11–14), revealing 6,798 genes, 6,711 coding sequences, 49 pseudogenes, 4 noncoding RNAs (ncRNAs), 16 frameshifted genes, 65 tRNA loci, and 87 RNA genes, which are comparable to other complete *B. cenocepacia* genomes (15–17).

Genes responsible for phosphate and nitrogen solubilization are present in the CR318 genome (2, 18–21). These genes agree with our results examining the plant growth-promoting characteristics of CR318 (F. Zekic, B. Weselowski, and Z.-C. Yuan, unpublished data). Further study of CR318 will allow for a greater understanding of the mechanisms underlying these characteristics, allowing the potential of CR318 to be exploited for agricultural applications.

### Accession number(s).

The complete sequence of *B. cenocepacia* CR318 has been deposited in NCBI’s GenBank with the accession numbers CP017238, CP017239, and CP017240. The version described in this paper is the first version.
